# The role and mechanism of pyroptosis and potential therapeutic targets in non-alcoholic fatty liver disease (NAFLD)

**DOI:** 10.3389/fcell.2024.1407738

**Published:** 2024-07-03

**Authors:** Shu-Jing Li, An-Bu Liu, Yuan-Yuan Yu, Jin-Hai Ma

**Affiliations:** ^1^ Department of Pediatrics Medical, General Hospital of Ningxia Medical University, Yinchuan, Ningxia, China; ^2^ Department of Emergency Medical, General Hospital of Ningxia Medical University, Yinchuan, Ningxia, China

**Keywords:** non-alcoholic fatty liver disease (NAFLD), pyroptosis, NOD-like receptor thermal protein domain associated protein 3(NLRP3) inflammasome, gasdermin D (GSDMD), caspase, targeted therapy

## Abstract

Non-alcoholic fatty liver disease (NAFLD) is a clinical pathological syndrome characterized by the excessive accumulation of fat within liver cells, which can progress to end-stage liver disease in severe cases, posing a threat to life. Pyroptosis is a distinct, pro-inflammatory form of cell death, differing from traditional apoptosis. In recent years, there has been growing research interest in the association between pyroptosis and NAFLD, encompassing the mechanisms and functions of pyroptosis in the progression of NAFLD, as well as potential therapeutic targets. Controlled pyroptosis can activate immune cells, eliciting host immune responses to shield the body from harm. However, undue activation of pyroptosis may worsen inflammatory responses, induce cellular or tissue damage, disrupt immune responses, and potentially impact liver function. This review elucidates the involvement of pyroptosis and key molecular players, including NOD-like receptor thermal protein domain associated protein 3(NLRP3) inflammasome, gasdermin D (GSDMD), and the caspase family, in the pathogenesis and progression of NAFLD. It emphasizes the promising prospects of targeting pyroptosis as a therapeutic approach for NAFLD and offers valuable insights into future directions in the field of NAFLD treatment.

## 1 Introduction

Non-Alcoholic Fatty Liver Disease (NAFLD) represents a clinical-pathological syndrome characterized primarily by excessive accumulation of fat within liver cells, diagnosed after excluding alcohol and other definitive hepatic damaging factors. This condition is closely associated with insulin resistance and genetic susceptibility, being considered an acquired metabolic stress-induced liver injury. NAFLD encompasses a spectrum of liver conditions primarily including simple fatty liver (SFL), non-alcoholic steatohepatitis (NASH), and associated cirrhosis (Pafili and Roden, 2021). As the prevalence of diabetes and obesity increases, the incidence of NAFLD has also risen. Recent statistics indicate that the global prevalence of NAFLD is approximately 20%–30% (Dewidar et al., 2020; Scorletti and Carr, 2022). SFL, as an initial stage of NAFLD, does not significantly impact liver function, and this process is reversible. NASH represents a progressive phase of NAFLD, characterized primarily by steatosis, inflammation, and fibrosis. The progression of NASH is irreversible and constitutes a major cause of end-stage liver disease (De Sant Ana et al., 2020). As the condition progresses, some patients with NASH may advance to liver fibrosis or even cirrhosis, ultimately leading to liver failure or hepatocellular carcinoma (Younossi et al., 2019; Zhou et al., 2021). This progression imposes a significant economic burden on both patients and healthcare systems (Younossi et al., 2019; Zhou et al., 2021). While advancements have been made in the diagnosis and management of NAFLD, existing managements largely focus on symptom management. Hence, delving into the molecular underpinnings of NAFLD holds significant promise for identifying novel therapeutic targets. Current research indicates that the pathogenesis of NAFLD is complex, with steatosis, lipotoxicity, and inflammatory responses playing critical roles (Cobbina and Akhlaghi, 2017). Recent studies have underscored the importance of pyroptosis, a specific type of cell death, as a pivotal element in modulating inflammation and immune responses (Yu et al., 2021; Li et al., 2023). Feng et al. 2022) observed that upregulation of phosphorylated STAT3 promotes Anxa2 expression at the transcriptional level, thereby activating caspase-1-mediated hepatocyte pyroptosis and fibrosis in NASH. Furthermore, the study involved the generation of high-fat diet (HFD)-induced WT and Casp11^−/−^ mice, followed by liver histopathological analysis, RNA-seq, FACS, Western blots, mitochondrial stress analysis in the hippocampus, and bone marrow transplantation. The research findings ultimately indicate that the caspase-11-GSDMD pathway-mediated cell pyroptosis plays a crucial role in promoting hepatic macrophage inflammation, thus contributing significantly to the development of NAFLD. Furthermore, this pathway holds promise as a potential therapeutic target for NAFLD (Drummer et al., 2023). Similarly, Canagliflozin acts by targeting the FGF21-ERK1/2 pathway to inhibit NLRP3-mediated pyroptosis, thus leading to an amelioration of NAFLD progression (Huang et al., 2023). These above investigations suggest that pyroptosis could have a significant impact on the onset and advancement of NAFLD.

Pyroptosis, alternatively termed inflammatory necrosis, represents a programmed cell death mechanism primarily distinguished by a profound inflammatory response. Key characteristics encompass pore formation on the cellular membrane, leading to cell swelling and subsequent rupture, with concurrent discharge of copious inflammatory mediators and cytoplasmic constituents. Pyroptosis is implicated in the onset and advancement of a multitude of inflammatory conditions (Xia et al., 2019; Xia et al., 2021). Previous studies have revealed that pyroptosis may play a critical part in hepatic pathology, particularly in the pathogenesis of NAFLD (de Carvalho Ribeiro and Szabo, 2022; Chen et al., 2023; Park et al., 2023). Indeed, moderate pyroptosis can facilitate the release of pathogens by eliminating infected cells, thus mobilizing immune cells to trigger the host immune response and safeguard the body against infection. However, excessive activation of pyroptosis may exacerbate inflammatory responses and trigger harm to cells or tissues, ultimately yielding adverse effects on prognosis. Research has demonstrated that uncontrolled NLRP3 activation has been shown to lead to shortened survival, severe liver inflammation, and activation of hepatic stellate cells (HSC), resulting in collagen deposition and liver fibrosis (Wree et al., 2018). Therefore, targeting hepatic cell pyroptosis to inhibit inflammation-induced liver damage can be considered a promising therapeutic approach for NAFLD. However, the underlying mechanism of NAFLD requires urgent and systematic investigation and interpretation. In this review, we will elucidate the key proteins and molecular mechanisms of pyroptosis, discuss the regulatory role of pyroptosis in NAFLD, and finally outline the current targeted-pyroptosis therapeutic strategies of NAFLD. This may provide a more comprehensive theoretical basis for the targeted therapy of NAFLD through pyroptosis.

## 2 Pyroptosis

Pyroptosis is a novel form of programmed cell death characterized by cell swelling followed by membrane rupture, leading to the release of cellular contents and activation of intense inflammatory responses (Wree et al., 2018; Wree et al., 2018). In 1992, Zychlinsky et al. (1992) first observed the phenomenon of pyroptosis. They described a lytic form of cell death in macrophages infected with *Shigella* bacteria. In 1996, Chen et al. (1996) reported that the invasion plasmid antigen B (IPAB) of *Shigella* flexneri can directly bind to ICE and activate ICE enzyme in infected macrophages. This form of cell death was initially thought to be apoptosis because some of its features resembled apoptosis, such as caspase dependency, DNA damage, and nuclear condensation. Subsequently, it was observed that this form of cell death is distinct from apoptosis. In 2001, D Souza and Heitman (2001) introduced the term “pyroptosis” to describe an inflammatory form of programmed cell death, marking the first definition of pyroptosis and distinguishing it from apoptosis. Currently, it is believed that pyroptosis primarily relies on the formation of membrane pores by members of the gasdermin (GSDM) protein family to release various inflammatory factors, such as IL-1 and IL-18 (Tang et al., 2019). Its hallmark is the formation of membrane pores, cell swelling, and rupture, accompanied by the release of numerous inflammatory factors and cellular contents (Cookson and Brennan, 2001; Galluzzi et al., 2018). When pyroptosis occurs, inflammatory cytokines and cellular contents are released from the cell after pore formation and cell lysis, leading to a pro-inflammatory cascade. Pyroptosis may play a dual role in the pathophysiological process. Specifically, moderate pyroptosis can recruit neutrophils to the site of infection to aid in capturing and clearing pathogens. This immune response targeting the pathogens is beneficial for eliminating infectious organisms. Nevertheless, excessive pyroptosis can trigger damage to the physical body. Therefore, the dynamic balance between inflammatory damage and pyroptosis-mediated immune responses should be finely regulated. When the aforementioned balance is disrupted, excessive host immune responses and cell death can result in serious disorders, such as hepatic lesions, sepsis, systemic lupus erythematosus (SLE), and others (Gautheron et al., 2020; Li et al., 2020; Li T. et al., 2022). Currently, multiple studies indicate that pyroptosis, lipotoxicity, and the innate immune response may trigger chronic inflammation in the liver, potentially fueling the transition from NAFL to NASH (Fredrickson et al., 2021; Nassir, 2022; Tacke et al., 2023). In the following, we will provide a detailed overview of the pyroptotic pathway, inflammasomes, and common signaling pathways to better elucidate the mechanism of pyroptosis in NAFLD.

### 2.1 Pathways of pyroptosis

Previous studies have indicated that the pyroptotic pathway can be divided into two categories based on the dependency on caspase-1: the caspase-1-mediated canonical pathway and the caspase-4, -5, or -11-mediated non-canonical pathway.

#### 2.1.1 The caspase-1-dependent pyroptotic pathway

The caspase-1-dependent pyroptotic pathway is as follows: Upon activation by various pathogen-associated molecular patterns (PAMPs) and damage associated molecular patterns (DAMPs), inflammasomes such as NOD-like receptor thermal protein domain associated protein 3(NLRP3), NLR Family CARD Domain Containing 4(NLRC4), absent in melanoma 2(AIM2), Pyrin, etc., can recognize these signals and become activated. Subsequently, they activate caspase-1 through the recruitment of the adaptor protein apoptosis-associated speck-like protein containing CARD (ASC) and binding with Pro-caspase-1. On one hand, activated caspase-1 cleaves Gasdermin D (GSDMD), exposing the N-terminus of GSDMD, which then binds to phospholipids on the cell membrane, forming pores that release cellular contents. The pores formed by GSDMD allow ions to enter the cell, leading to changes in osmotic pressure, cell swelling, cell rupture, accompanied by the release of cellular contents, ultimately resulting in pyroptosis. On the other hand, activated caspase-1 cleaves and activates the precursors of IL-1β and IL-18. The activated IL-1β and IL-18 are then released into the extracellular space, amplifying the inflammatory response.

#### 2.1.2 The caspase-1-independent pyroptotic pathway

**FIGURE 1:**
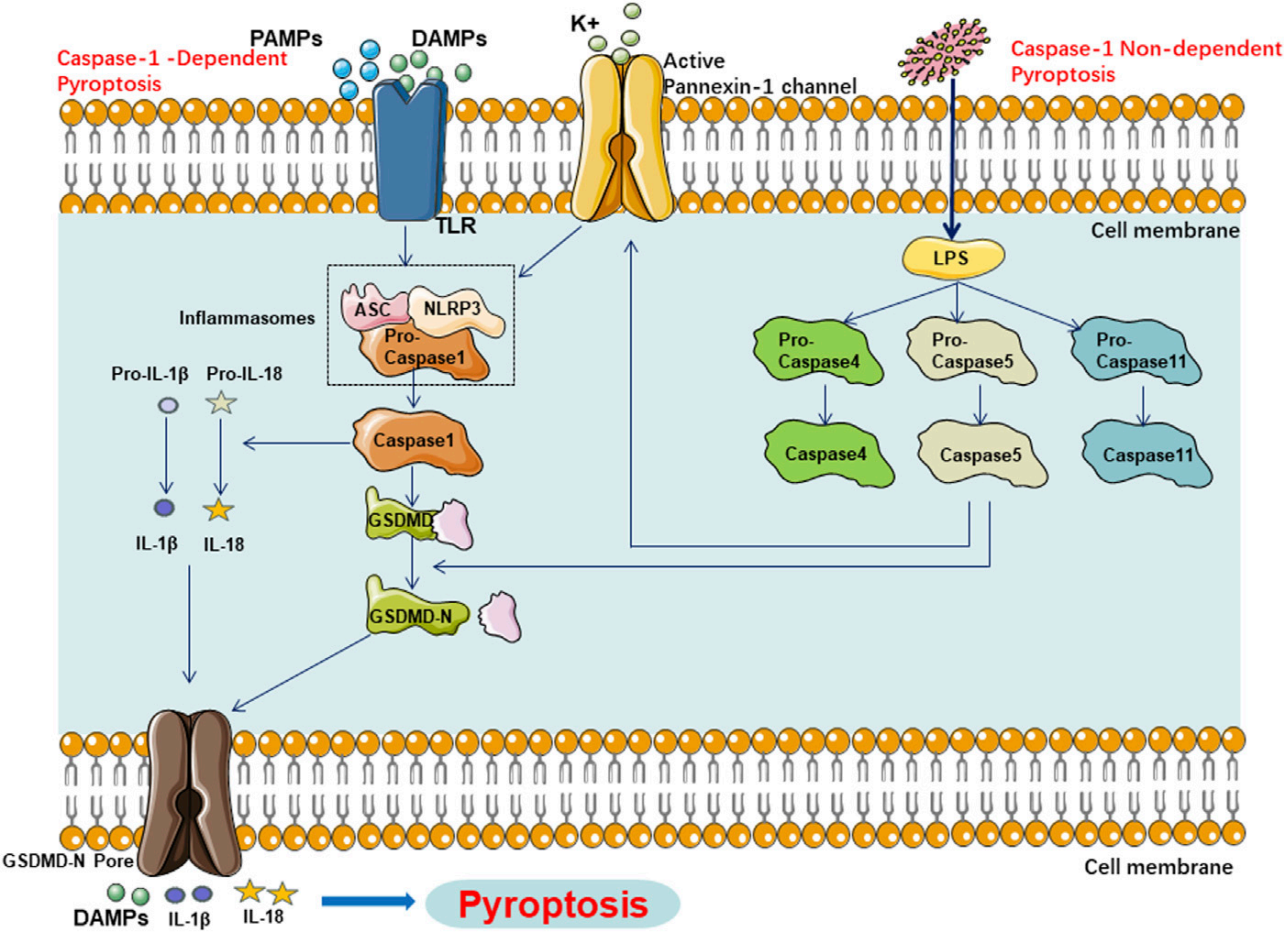
Main Pathway of Pyroptosis in NAFLD, Pyroptosis in NAFLD can be categorized into two types depending on whether they are caspase1 dependent or not. In caspase1 dependent pyroptosis, the process is initiated by the assembly of inflammasomes. In caspase-1 non-dependent pyroptosis can be triggered by the interaction between caspase-4, caspase-5, or caspase-11 and LPS.

Moreover, the caspase-1-independent pyroptotic pathway is as follows: Upon stimulation by lipopolysaccharide (LPS), caspase-4, caspase-5, and caspase-11 can directly bind to LPS and become activated. They directly cleave the GSDMD protein, exposing its N-terminus and initiating the pyroptotic process. Additionally, the activated caspase-4/5/11 activates the Pannexin-1 channel, releasing K+ into the extracellular space, which activates the NLRP3 inflammasome, inducing the activation of caspase-1 and further activating the caspase-1-dependent pyroptotic pathway. This illustrates the close connection between the canonical and non-canonical pyroptotic pathways (Figure1).

### 2.2 Pyroptosis key proteins

#### 2.2.1 NLRP3 inflammasome

The inflammasome is a complex formed by multiple intracellular proteins. It acts as a cytosolic sensor receiving activation signals through nucleotide-binding oligomerization domain (NOD)-like receptors (NLRs), comprising nucleotide-binding oligomerization domain, NLR, bridging adapter, and effector components. Indeed, the assembly of the inflammasome mainly involves pattern recognition receptors (PRRs), the apoptosis-associated speck-like protein containing a caspase recruitment domain (ASC), and caspase-1. Obviously, there are four main types of PRRs involved in pathogen recognition: Toll-like receptors (TLRs), Nod-like receptors (NLRs), RIG-I-like receptors (RLRs), and C-type lectin receptors. These PRRs can recognize conserved microbial structural units or PAMPs such as peptidoglycans. They can also identify DAMPs generated by cellular or tissue injury, such as adenosine triphosphate (ATP). In addition, TLRs are typically located on the cell’s external surface and endosomes, while NLRs are positioned in the cytoplasm, recognizing PAMPs and DAMPs that enter the intracellular environment. Activated inflammasomes can detect DAMPs produced by damaged cells and PAMPs from pathogens in the gut-liver axis. They assemble into complexes to induce the activation of caspase-1, participating in the caspase-1-dependent cell pyroptosis pathway. To date, five PRRs have been identified to form inflammasomes, including NLRP1, NLRP3, NLRC4, Pyrin, and AIM2, which are believed to play crucial roles in hepatic inflammation, fibrosis, and other pathological conditions (Zhang W.-J. et al., 2021). Furthermore, the NLRP3 inflammasome has been demonstrated to play a critical role in amplifying hepatic inflammation, activating immune cells, and triggering hepatic cell injury. Hence, the NLRP3 inflammasome is considered a crucial player in hepatic disorders, including NAFLD, where pyroptosis is involved. Targeting the NLRP3 inflammasome for the management of NAFLD holds great promise for the future.

The NLRP3 inflammasome is a large multi-protein complex with a relative molecular mass of approximately 700,000 Daltons, consisting of NLRP3, the adaptor protein ASC, and the effector protein caspase-1. Assembly of the NLRP3 inflammasome requires interactions between the NLRP3 receptor, ASC adapter, and pro-caspase-1. NLRP3 belongs to the NLR protein family. The structure of most NLRs is similar, and NLRP3 consists of three main parts: the N-terminal caspase recruitment domain (CARD), the pyrin domain (PYD), and the acidic transactivation domain or the baculovirus inhibitor of apoptosis protein repeat domain (BIR), which mainly mediate interactions with downstream proteins. The second part is the central nucleotide-binding and oligomerization domain (NACHT), which is involved in self-oligomerization and includes proteins such as NAIP, CIITA, HET-E, and TP1. The third part is the C-terminal leucine-rich repeats (LRRs), which are primarily responsible for recognizing stimuli. Under physiological conditions, the NACHT domain of NLRP3 can bind to LRRs, maintaining a self-inhibited state. When PAMPs or DAMPs are present, NLRP3 is released from self-inhibition, exposing the NACHT domain, which facilitates oligomerization and serves as a scaffold. The N-terminal PYD domain of NLRP3 recruits the ASC adaptor protein containing a PYD. The CARD domain of ASC recruits pro-caspase-1 containing a CARD, thereby completing the assembly of the inflammasome.

The adaptor protein ASC consists of PYD and CARD domains. ASC is primarily located in the nucleus of human monocytes/macrophages and rapidly translocates to the cytoplasm upon stimulation. It links NLRP3 and pro-caspase-1, boosting the activation of the NLRP3 inflammasome.

Caspase-1, also known as the IL-1β-converting enzyme, serves as the effector protein of the NLRP3 inflammasome. It is produced by self-cleavage of the pro-caspase-1 precursor molecule, leading to the formation of active caspase-1. Its primary role is to cleave pro-IL-1β and pro-IL-18 into their mature forms, IL-1β and IL-18.

#### 2.2.2 GSDMD

The GSDMD protein belongs to the gasdermin (GSDM) family and is a key contributor to pyroptosis. Research indicates that the human and mouse genomes encode 6 members (GSDMA, GSDMB, GSDMC, GSDMD, GSDME, DFNB59) and 10 members (GSDMA1, GSDMA2, GSDMA3, GSDMC, GSDMC2, GSDMC3, GSDMC4, GSDMD, GSDME, DFNB59) of the GSDM protein family, respectively. Similar to other family members, the GSDMD protein comprises a cytotoxic N-terminal domain and a C-terminal inhibitory domain connected by a flexible linker. Upon cleavage of the C-terminal domain of GSDMD, the GSDMD-N terminal is recruited to interact with lipids in the cell membrane, generating an intermediate structure called a pre-pore without membrane insertion. Following a conformational rearrangement, the pre-pore transforms into a curved oligomer, which then progresses into a fissile form, ultimately growing into a circular oligomer and forming a membrane pore (Aglietti et al., 2016). Moreover, the electron microscopy reveals that the inner diameter of the GSDMD-N pore is 10–15 nm, allowing certain pro-inflammatory cytokines such as IL-1β and IL-18 to pass through, triggering pyroptosis (Barnett and Ting, 2020; Xiaodong and Xuejun, 2023). It is worth noting that the transcription of GSDMD can be regulated by multiple molecules. For instance, in adipocytes, NF-κB can activate the transcription of GSDMD (Jin et al., 2022). In endothelial or macrophage cells, the activation of IRF1/2 can enhance the expression of GSDMD (Benaoudia et al., 2019; Kayagaki et al., 2019). As a common effector of the inflammasome, inhibition of GSDMD cleavage and oligomerization can block its role in cell pyroptosis, thereby achieving the therapeutic goal of treating diseases.

#### 2.2.3 Caspase family

The caspase family comprises cysteine proteases that play pivotal roles in cell death and inflammatory responses by recognizing substrates and cleaving after aspartate (D) residues to activate substrate proteins. In the absence of upstream signals, caspase family members exist in the cytoplasm as inactive proenzymes, and substrate recognition and cleavage occur only after caspases undergo self-cleavage activation in response to upstream signals. There are currently 14 types of caspases discovered, classified into three categories based on their involvement in distinct cellular physiological processes: apoptosis-related caspases (caspase-2, 8, 9, 10, 3, 6, 7), pyroptosis-related caspases (caspase-1, 4, 5, 11, 12), and other caspases (caspase-14). Caspase-1 is involved in the classical cell pyroptosis pathway, while caspase-4/5/11 mediate the non-classical pyroptosis pathway induced by LPS. Previous studies have revealed that caspase-3 and caspase-8 can also cleave the GSDMs family, thereby participating in cell pyroptosis pathways (Wang et al., 2017; Fritsch et al., 2019). This may indirectly suggest a certain connection between pyroptosis and apoptosis.

## 3 The role of pyroptosis in NAFLD

NAFLD is a refractory chronic hepatic disease characterized by the accumulation of fat in liver cells leading to inflammation. Chronic fat accumulation in hepatic cells can progress from simple steatosis to steatohepatitis, triggering irreversible hepatic damage and accelerating hepatic fibrosis. Currently, researches have revealed the involvement of multiple metabolic regulatory mechanisms, cellular death pathways, inflammation activation, oxidative stress, and other factors in the initiation and progression of NAFLD (Lin et al., 2020; Loomba et al., 2021). However, there is no definitive conclusion yet regarding the molecular mechanisms underlying the development of NAFLD. Currently, multiple studies have indicated that the inflammatory response and inflammation-characterized pyroptosis can be closely associated with the occurrence and development of NAFLD (Pan et al., 2018; Koh et al., 2021). Wree et al. (2014a) generated global and myeloid cell-specific conditional mutant NLRP3 knock-in mice expressing the D301N NLRP3 mutation (orthologous to D303N in human NLRP3), resulting in hyperactive NLRP3. The study found that overactivation of NLRP3 can lead to shortened survival, growth retardation, and severe hepatic inflammation in mice (Wree et al., 2014a). Additionally, a clinical trial found that levels of full-length GSDMD and cleaved GSDMD-N protein in the liver were higher in NAFLD/NASH patients compared to the normal population (Xu et al., 2018). Besides, the study also revealed that levels of GSDMD-N protein in the liver of human NASH patients were significantly elevated and correlated with NAFLD activity scores and fibrosis (Xu et al., 2018). Therefore, GSDMD-N could serve as a potential biomarker for diagnosing NASH (Xu et al., 2018). After hepatocytes undergo pyroptosis, the cell membrane ruptures, releasing a significant amount of inflammatory mediators, thereby accelerating the progression of hepatic inflammation and fibrosis. Consequently, we will further elaborate on the role of pyroptosis in NAFLD focusing on the three key proteins: NLRP3 inflammasome, GSDMD, and caspases.

### 3.1 NLRP3 inflammasome in NAFLD

#### 3.1.1 Activation of NLRP3 inflammasome

NLRP3 inflammasome activation can boost the activation of pro-caspase-1 and the release of key inflammatory factors, which is vital for the initiation and development of NAFLD. The activation of the NLRP3 inflammasome occurs in two steps. The first step involves microbial or endogenous molecules inducing nuclear factor-κB (NF-κB) translocation, leading to upregulation of NLRP3 and pro-IL-1β transcription. The second step is triggered by ATP, pore-forming toxins, viral RNA, or particulate matter, which, through various cellular and molecular effects, promote inflammasome assembly and mediate the cleavage of pro-IL-1β into its mature form. In the pathogenesis of NAFLD, the activation of NLRP3 inflammasome in the second step can be triggered by various intracellular signals, including extracellular ATP, cholesterol crystals, P2X purinergic receptor 7, mitochondrial ROS, mitochondrial DNA (mtDNA), uric acid, cardiolipin, perfluorooctanoic acid (PFOA), endoplasmic reticulum (ER) stress, and impaired mitochondrial autophagy. Next, we will proceed with individual introductions.

##### 3.1.1.1 The role of ATP-citrate lyase (ACLY) in activation of NLRP3 inflammasome

ATP-citrate lyase (ACLY) is an enzyme situated upstream of ACC within the TCA cycle, capable of converting citrate derived from the tricarboxylic acid (TCA) cycle into acetyl-CoA and oxaloacetate in the cytoplasm. The acetyl-CoA produced in the cytoplasm can be transformed into malonyl-CoA by ACC or converted into cholesterol by hydroxymethylglutaryl-CoA (HMG-CoA) synthase. ACLY has been demonstrated to be an epigenetic driver promoting the progression of NAFLD (Morrow et al., 2022). Further mechanistic studies have revealed that ACLY plays a crucial role in the activation of the NLRP3 inflammasome, characterized by the following observations: inhibition of hepatic ACLY leads to an increase in fatty acid oxidation in liver cells, a reduction in fatty acid and sterol synthesis, and a mitigation of hepatic steatosis and ballooning. These findings suggest that ACLY may serve as an effective target for the management of NASH and dyslipidemia (Morrow et al., 2022).

##### 3.1.1.2 The role of free cholesterol in hepatocytes in activation of NLRP3 inflammasome

Moreover, an overload of free cholesterol in hepatocytes can lead to endoplasmic reticulum stress, mitochondrial dysfunction, the production of reactive oxygen species (ROS), and the formation of cholesterol crystals in lipid droplets. These events subsequently trigger the activation of the NLRP3 inflammasome, ultimately inducing pyroptosis. Pyroptosis of hepatic cells and cholesterol overloading can activate Kupffer cells and hepatic stellate cells, leading to the development of NASH and hepatic fibrosis (Farrell et al., 2018; Horn et al., 2022).

##### 3.1.1.3 The role of P2X purinoceptor 7 (P2RX7) in activation of NLRP3 inflammasome

The P2RX7 has been recognized as a key facilitator in the activation of the NLRP3 inflammasome and the processing of IL-1β, with its involvement corroborated through mouse models subject to dietary and chemically induced liver damage (Luo et al., 2022). It is of significant interest that recent research has unveiled extracellular ATP, functioning as an intracellular danger signal, directly triggers the plasma channel through P2RX7, resulting in the influx of K+ into the cell (Rossi et al., 2022). This serves as a pivotal direct catalyst for the activation of the NLRP3 inflammasome. Bernat Baeza-Raja and colleagues further discovered P2RX7 expression in mononuclear cells and Kupffer cells within the livers of NASH patients. Additionally, their research verified that pharmacologically inhibiting P2RX7 in these cell types can inhibit IL-1β secretion, decrease hepatic caspase 3/7 activity, and consequently reduce cell pyroptosis facilitated by these enzymes (Baeza-Raja et al., 2020).

##### 3.1.1.4 The role of ROS in activation of NLRP3 inflammasome

ROS is considered a common signal for NLRP3 inflammasome activation, playing a significant role in the onset and progression of hepatic steatosis and NASH. Interestingly. Besides, lipid metabolism disorder is a prominent feature in the pathological development of NAFLD. Specifically, the abnormal accumulation of free fatty acids within hepatocytes triggers increased ROS production, accelerating hepatic steatosis (Marra and Svegliati-Baroni, 2018). Hence, targeting ROS to suppress the activation of the NLRP3 inflammasome and thus treat NAFLD may be feasible. For example, in a mouse model of NAFLD induced by a high-fat diet (HFD), Danshen zexie decoction (DZD) can suppress ROS production in the rats, upregulate SOD activity and GSH levels, while decreasing serum levels of IL-1β and IL-18. Ultimately, this intervention inhibited the expression of NLRP3, caspase-1, GSDMD, and GSDMD-N (Biao et al., 2022). Furthermore, Gardenoside can effectively reduce lipid accumulation in NAFLD, enhance cell viability, decrease ROS production, and alleviate pyroptosis in both *in vitro* and *in vivo* NAFLD models (Shen et al., 2021).

##### 3.1.1.5 The role of mitochondrial autophagy in activation of NLRP3 inflammasome

Importantly, it should not be overlooked that the NLRP3 inflammasome triggered by impaired mitochondrial autophagy is a critical juncture in the progression from NAFLD to NASH (Zhang et al., 2019). This indirectly indicates that autophagy and pyroptosis interact synergistically in contributing to the occurrence and progression of NAFLD.

##### 3.1.1.6 The role of perfluorooctanoic acid (PFOA) in activation of NLRP3 inflammasome

Furthermore, clinical studies have shown an association between perfluorooctanoic acid (PFOA) and the development of NAFLD (E et al., 2023). Further investigation into the potential mechanisms of PFOA in NAFLD reveals that exposure to PFOA in mice can result in lipid accumulation and stimulate lipid generation in the mouse liver and L-02 cells. Additionally, increased NLRP3 aggregation and enhanced IL-1β production were detected, ultimately leading to pyroptosis (Weng et al., 2020).

##### 3.1.1.7 The role of uric acid in activation of NLRP3 inflammasome

Clinical studies have shown that a high-sugar and high-fat diet can lead to elevated serum uric acid levels, increased triglyceride concentrations in the liver, and a positive correlation with the incidence of NAFLD (Muriel et al., 2021). Uric acid can trigger NLRP3 inflammasome activation, thereby regulating hepatic steatosis and insulin resistance. Therefore, uric acid may be a new target for the treatment of NAFLD and insulin resistance (Wan et al., 2016). In addition to treating hyperuricemia, previous studies have found that xanthine oxidase can regulate the activation of the NLRP3 inflammasome, thereby targeted-treating NAFLD (Xu et al., 2015). Similarly, Liraglutide can attenuate uric acid levels, suppress the activation of the NLRP3 inflammasome, and thereby alleviate NAFLD in rats (Ma et al., 2022).

##### 3.1.1.8 The role of cardiolipin in activation of NLRP3 inflammasome

Cardiolipin is primarily located in the inner mitochondrial membrane of animal cells, with 15% found in cardiac muscle. It has been reported that cardiolipin effectively triggers the activation of the NLRP3 inflammasome through an ROS-independent signaling pathway, contributing to the development of NASH (Peng et al., 2018; Ramos-Tovar and Muriel, 2023). In a NASH mouse model, cardiolipin synthase 1 (CLS-1) inhibits the synthesis of cardiolipin, thereby suppressing the activation of the NLRP3 inflammasome and relieving NASH (Liu et al., 2019).

#### 3.1.2 The role of activated NLRP3 inflammasome in NAFLD

The activated NLRP3 inflammasome might be vital in the initiation and progression of NAFLD. Here, we might primarily elucidate the role of NLRP3 and caspase-1 in NAFLD through the activation of the NLRP3 inflammasome.

##### 3.1.2.1 The role of NLRP3 in the occurrence and progression of NAFLD

###### 3.1.2.1.1 The abnormal activation of NLRP3 is positively correlated with NAFLD

Wree et al. (2014b) generated loss-of-function and gain-of-function NLRP3 mouse models, and confirmed in these models that NLRP3 deficiency has a protective effect against diet-induced steatohepatitis, hepatomegaly, liver injury, and activated macrophage infiltration. Currently, the prevailing view suggests that NLRP3 is highly expressed in hepatocytes, macrophages, and hepatic stellate cells. Its activation can significantly exacerbate NAFLD and promote the secretion of pro-inflammatory cytokines IL-1β and IL-18 (Swanson et al., 2019). However, A study suggest that NLRP3 may negatively regulate the progression of NAFLD/NASH by modulating the gut microbiota (Csak et al., 2011). For example, Pierantonelli et al. found that compared to wild-type mice, mice lacking the NLRP3 gene exhibited more severe hepatic triglyceride accumulation and steatosis after being fed a high-sugar, high-fat diet. The speculated reason may be that the absence of the NLRP3 gene leads to immune dysregulation in the intestines, increased intestinal permeability, dysbiosis of beneficial bacteria, resulting in bacterial translocation, and increased expression of liver TLR4 (LPS receptor) and TLR9 (dsDNA receptor) (Gaul et al., 2021). In comparison to non-NASH patients, NASH patients exhibit significantly higher levels of NLRP3, caspase-1, proIL-1β, and pro-IL-18 in liver samples, and their expression is associated with the development/severity of fibrosis (Shi et al., 2023). Susanne Gaul et al. detected activated forms of caspase-1 in the liver and serum of NASH patients, which were correlated with the severity of the disease (Cai et al., 2017). Moreover, Empagliflozin participates in the treatment of NASH by inducing ASC oligomerization to suppress NLRP3 inflammasome activation and inhibit caspase-1 (Van Rooyen et al., 2011).

###### 3.1.2.1.2 The aberrant activation of NLRP3 promotes an increase in collagen activity in the hepatic inflammatory zone

Moreover, current molecular studies further confirm the direct involvement of activated NLRP3 inflammasomes in NASH and NAFLD. For instance, Alexander Wree et al. (2014a) observed that NLRP3 knock-in in mouse hepatocytes significantly increased caspase-1 expression, leading to increased collagen deposition in the hepatic inflammatory areas. Additionally, the study detected the release of two common liver injury markers, connective tissue growth factor (CTGF) and tissue inhibitor of metalloproteinase 1 (TIMP1), from hepatic stellate cells, further confirming that aberrant activation of NLRP3 induces hepatocyte pyroptosis, resulting in severe hepatic inflammation, hepatic stellate cell activation, and collagen deposition.

Kupffer cells (KCs) are liver-resident macrophages responsible for containing and clearing harmful exogenous particles and immune reactants. Pan et al. (2018) discovered that palmitic acid (PA), acting as a DAMP, stimulates KCs to induce the release of mitochondrial DNA (mtDNA) from mitochondria to the cytosol. The released mtDNA directly binds to the NLRP3 inflammasome, leading to the activation of caspase-1 and the release of pro-inflammatory IL-1β, thereby exacerbating NASH. Similarly, the deletion of the NLRP3 gene in mice has been shown to alleviate palmitic acid-induced KC inflammation and halt the progression of NASH (Gallego et al., 2020). Similar results were obtained in another high-fat diet (HFD)-induced NAFLD mouse model (Henao-Mejia et al., 2012). Furthermore, in mice with NLRP3 gene knock-in, even after 9 months of normal diet, there was an increase in the expression levels of hepatic fibrosis markers. This was accompanied by increased collagen deposition and elevated expression of alpha-smooth muscle actin (α-SMA) protein (Cai et al., 2017). Therefore, it can be confirmed that excessive activation of NLRP3 leads to hepatocyte pyroptosis, resulting in the secretion of inflammatory complexes into the extracellular space, thereby activating hepatic stellate cells (HSCs) and promoting collagen production. Similarly, Gallego et al. found that the absence of NLRP3 can modulate the process of liver fibrosis, alleviating age-related hepatic fibrosis pathology during the aging process (Pierantonelli et al., 2017). In summary, the prevailing consensus is that the aberrant activation of NLRP3 is positively associated with NAFLD.

###### 3.1.3.1 The role of caspase-1 in the occurrence and progression of NAFLD

Another crucial component in the NLRP3 inflammasome, caspase-1, is also involved in NAFLD. A recent study has indicated that caspase-1 can activate pro-IL-1β, promoting the accelerated development of NAFLD into its active form, IL-1β (Morrison et al., 2016). The application of caspase-1 inhibitors can alleviate the progression of NASH, liver fibrosis, and insulin resistance (Morrison et al., 2016). Furthermore, caspase-1/11 can modulate the composition and diversity of the gut microbiota, thereby influencing hepatic lipid composition and metabolism, and facilitating the progression of NAFLD (De Sant Ana et al., 2020).

### 3.2 GSDMD in NAFLD

GSDMD acts as the ultimate executioner of pyroptosis, being cleaved by caspase enzymes to generate a pore-forming N-terminal fragment, which plays a role in the initiation and development of NAFLD. Current research indicates that GSDMD may play a role in the occurrence and progression of NAFLD by regulating lipid metabolism and the secretion of pro-inflammatory cytokines.

#### 3.2.1 The involvement of GSDMD in regulating lipid metabolism in NAFLD

On the one hand, GSDMD may be positively associated with hepatic steatosis. Xu et al. found that compared to wild-type mice, GSDMD^−/−^ mice fed with a methionine-choline deficient (MCD) diet exhibited lower levels of steatosis (Xu et al., 2018). On the other hand, GSDMD may exert an influence on hepatic lipid synthesis by modulating the expression of lipid-related genes. It was observed that the absence of the GSDMD gene in mice resulted in a reduction in the expression of the lipogenic gene Srebp-1c, while the expression of the lipolytic gene Pparα and its downstream targets Aco, Lcad, Cyp4a10, and Cyp4a14 increased. Additionally, significantly elevated levels of hepatic GSDMD-N protein in human NASH patients compared to the non-NASH control group, showing associations with NAFLD activity scores and fibrosis (Xu et al., 2018). Hence, GSDMD-N holds promise as a potential diagnostic biomarker for NASH.

#### 3.2.2 The role of GSDMD in regulating the secretion of pro-inflammatory cytokines in NAFLD

Significantly, GSDMD can modulate the pathophysiological processes of NAFLD by regulating the secretion of pro-inflammatory cytokines. Previous studies have indicated that the elimination of GSDMD leads to a substantial decrease in the hepatic production of pro-inflammatory cytokines including monocyte chemoattractant protein-1 (MCP-1), TNF-α and IL-1β (Dela Peña et al., 2005; Xu et al., 2018). For instance, MCP-1 triggers hepatic macrophage infiltration and lipid accumulation by diminishing the secretion of apolipoprotein B and augmenting the messenger RNA levels of phosphatidylinositol 3-kinase (Clément et al., 2008). Moreover, individuals with NAFLD may exhibit red blood cell-mediated secretion of the chemotactic factor MCP-1, resulting in heightened TNF-α release by macrophages (Papadopoulos et al., 2022). Thus, it can be inferred that MCP-1 plays a direct role in the stimulation and recruitment of macrophages. TNF-α might play a pivotal role in the pathogenesis of diet-induced NASH by activating inflammatory cells to exert its significant effects (Kugelmas et al., 2003; Zhang et al., 2023). Similarly, IL-1β can promote the proliferation and transdifferentiation of hepatic stellate cells and is responsible for the accumulation of fibrotic tissue. Previous studies have found that IL-1β promotes hepatic cell proliferation associated with NASH (Kucsera et al., 2023). In addition to the aforementioned studies, a meta-analysis has revealed a significant association between elevated concentrations of certain inflammatory mediators, including IL-1β, IL-6, TNF-α, and ICAM-1, and an inclined risk of NAFLD (Duan et al., 2022). In conclusion, it can be inferred that GSDMD primarily promotes the occurrence and progression of NAFLD through the regulation of lipid metabolism and the secretion of pro-inflammatory cytokines.

Based on the above research, GSDMD represents a promising therapeutic target for NAFLD. Several medications have been identified to target GSDMD for NAFLD treatment. For example, Jiangzhi Ligan Decoction (JZLGD) has demonstrated hepatoprotective effects in a rat model of NAFLD induced by a high-fat diet by regulating the GSDMD-mediated canonical/non-canonical pyroptosis pathway (Yin et al., 2021). Si-Wu-Tang has shown efficacy in inhibiting the TLR4-JNK and caspase-8-GSDMD signaling pathways, offering therapeutic benefits for NAFLD (Zhang et al., 2020). Similarly, Baicalin (BA) exhibits promise in reducing NLRP3 and GSDMD expression levels, as well as the secretion of IL-1β and IL-18, through modulation of the NRF2/HO-1/NRLP3 pathway, thereby alleviating pyroptosis in hepatic cells of NASH patients (Shi et al., 2020; Shi et al., 2022).

### 3.3 Caspase family in NAFLD

Having elucidated the pivotal roles of the NLRP3 inflammasome and GSDMD in NAFLD, it is pertinent to investigate the implication of the caspase family in pyroptosis within this context. Fundamentally, inflammatory caspases are accountable for instigating the activation of IL-1β and IL-18, consequently contributing to the progression of pyroptosis in NAFLD. Gaul et al. have substantiated the presence of the caspase-1-mediated canonical pyroptosis pathway in hepatic cells (Shi et al., 2022). Consistent results have been noted across various dietary inductions. In a murine model of NASH induced by a high-fat, high-cholesterol diet (HFHCD), the deficiency of caspase-1 has demonstrated efficacy in averting liver inflammation, fibrosis, and hepatic cell pyroptosis (Koh et al., 2021). Moreover, the non-classical pathway mediated by caspase-11 has been implicated in the progression of hepatic steatosis and NASH. Charles Drummer et al. (2023) revealed that a high-fat diet (HFD) can upregulate the expression of caspase-11, GSDMD and interleukin-1β in wild-type (WT) mice, while downregulating pyroptosis in monocyte-derived macrophages (MDM) and surface GSDMD expression in inflammatory monocytes (IM). This cascade ultimately leads to reduced hepatic lipid accumulation and NAS score. Moreover, elevated levels of serum ALT, AST, and hepatic triglycerides have been observed in mice overexpressing caspase-11, concomitant with exacerbated hepatic steatosis and deteriorated liver injury (Zhu et al., 2021). Subsequent investigations have revealed that caspase-1/11 is found to exert influence on the gut microbiota, thereby impacting the progression of NAFLD (Arsenijevic et al., 2019). In an animal study, caspases 1/11 knockout mice were found to be more susceptible to HFD-induced hepatic steatosis compared to their wild-type counterparts (Arsenijevic et al., 2019). Furthermore, elevated levels of Proteobacteria and an increased Firmicutes/Bacteroidetes ratio were observed in the knockout mice (Arsenijevic et al., 2019). Consequently, it can be inferred that mice deficient in caspases 1/11 hosted gut bacterial phyla linked to hepatic steatosis (Arsenijevic et al., 2019). The aforementioned investigations indicate that caspase-1/11 exerts a crucial influence on the modulation of both hepatic lipid composition and gut microbiota composition, implicating its involvement in the development of NAFLD. It may highlight the potential of caspase-1/11 as a promising therapeutic target for intervention in NAFLD. For instance, inulin can exert beneficial effects on NAFLD by modulating the gut microbiota and inhibiting the lipopolysaccharide-toll-like receptor 4-myeloid differentiation primary response 88-nuclear factor-kappa B-nucleotide-binding oligomerization domain-like receptor protein 3 pathway along the gut-liver axis in mice (Bao et al., 2020). Similarly, lycopene demonstrates therapeutic effects on NAFLD in high-fat high-fructose diet-fed mice by declining the levels of pro-caspase-1 and caspase-1 in the liver, thereby modulating the NF-κB/NLRP3 inflammasome pathway and gut microbiota composition (Gao et al., 2023).

**FIGURE 2:**
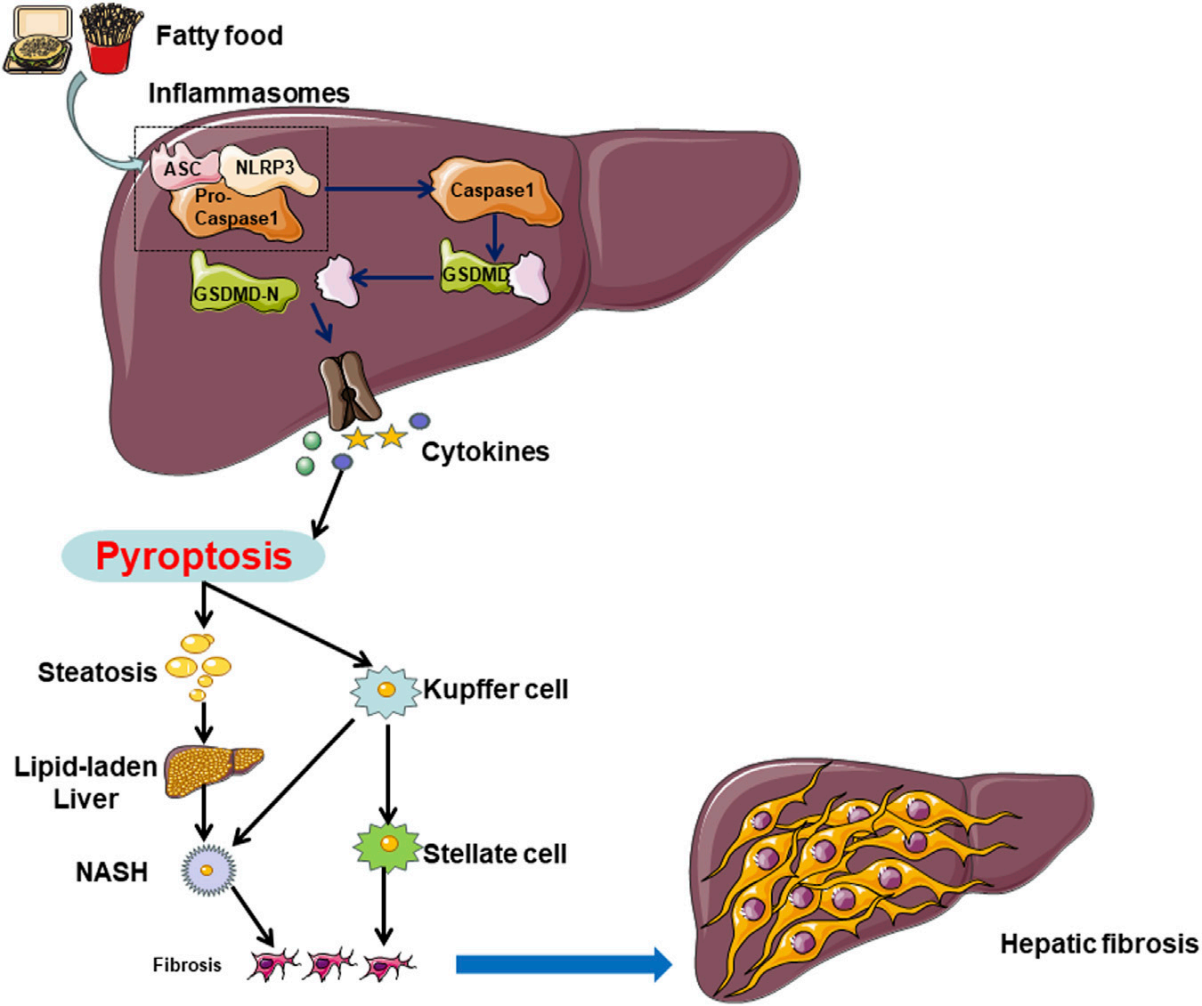
In NAFLD, hepatocytes can undergo cell death through both caspase-1-dependent and non-caspase-1-dependent pathways. Multiple factors, including a high-fat diet and unhealthy lifestyle choices, can induce hepatocyte pyroptosis in the liver. Hepatocyte cell pyroptosis facilitates lipid accumulation by inhibiting lipid breakdown and promoting lipid deposition, leading to lipid degeneration. On the other hand, hepatocyte pyroptosis releases NLRP3 inflammasomes, which are internalized by Kupffer cells (KCs) and hepatic stellate cells (HSCs), inducing pyroptosis in each cell type. Pyroptosis in hepatocytes and KCs promotes the transition from lipid degeneration to non-alcoholic steatohepatitis (NASH), while pyroptosis in HSCs leads to the progression from NASH to liver fibrosis.

In summary, the pivotal molecular players implicated in pyroptosis, such as the NLRP3 inflammasome, GSDMD, and caspases, exert a critical influence on the initiation and advancement of NAFLD by contributing to pyroptosis and associated inflammatory reactions within hepatocytes, Kupffer cells, and hepatic stellate cells. (Figure 2) These molecules represent potential therapeutic targets for the future management of NAFLD.

## 4 Targeted therapy in NAFLD

As previously elaborated, the significance of NLRP3 inflammasome, GSDMD, and the caspase family in orchestrating pyroptosis has been firmly established in the etiology and advancement of NAFLD and NASH. These discoveries can lay a robust theoretical groundwork for directing interventions towards pyroptosis as a promising therapeutic strategy for managing NAFLD. Given the intricate nature of the NLRP3 inflammasome signaling pathway implicated in pyroptosis, the activation of diverse target proteins can be restrained through a variety of mechanisms. These can include blocking upstream signals, inhibiting inflammasome assembly, suppressing caspase-1 activation, preventing GSDMD cleavage, and halting the production of inflammatory cytokines (Zahid et al., 2019). Prior research has suggested that Liraglutide, an analog of glucagon-like peptide-1, exhibits potential in the treatment of type 2 diabetes and prevention of NASH by inhibiting the activation of the NLRP3 inflammasome (Armstrong et al., 2016; Yu et al., 2019; Ao et al., 2020; Ma et al., 2022). In HepG2 cells, liraglutide binds to toll-like receptors, exerting inhibitory effects on the upregulation of NLRP3 and proIL-1β, as well as the activation of inflammasomes and pyroptosis. These actions ultimately contribute to the delaying of NASH progression (Yu et al., 2019). It is noteworthy that MCC950, classified as a small molecule compound, has been unequivocally identified as an NLRP3 inhibitor (Li H. et al., 2022). MCC950 exerts its effects by inhibiting the activation of the NLRP3 inflammasome and the production of IL-1β through the inhibition of ASC oligomerization. Further molecular mechanistic insights have indicated that MCC950 can directly interact with the Walker B motif located within the NACHT domain of NLRP3, leading to the disruption of ATP hydrolysis, thereby inhibiting NLRP3 activation and the assembly of the inflammasome (Coll et al., 2019). In addition to liraglutide and MCC950, specific constituents of traditional herbal medicine have been demonstrated to play a critical role in NAFLD treatment. For example, Scutellarin, a natural plant-derived flavonoid, has exhibited the ability to inhibit the activation of NLRP3 inflammasomes in macrophages, thereby enhancing protein kinase A signaling for protective effects in mice (Liu et al., 2017). In a murine model of NAFLD, silymarin suppresses NLRP3 inflammasome activation via the nicotinamide adenine dinucleotide (NAD+)/SIRT2 pathway (Zhang B. et al., 2018). Onions are rich in dihydroflavonols, which mitigate alcoholic liver steatosis by decreasing NLRP3 levels and suppressing the production and release of IL-1β (Zhang Y. et al., 2018). In addition to the aforementioned therapeutics, a multitude of drugs aimed at targeting pyroptosis and pivotal molecules linked to pyroptosis are presently under development for precise treatment of NAFLD (Table 1). Although the majority of these drugs are currently in the early stages of preclinical investigation and lack comprehensive large-scale clinical validations, extensive research remains imperative in the future to assess the effectiveness and safety profiles of such pharmaceutical agents. Nonetheless, the substantial therapeutic potential of directing focus towards the NLRP3 inflammasome and its associated pyroptotic pathway in the management of NAFLD warrants considerable attention.

**TABLE 1:** Therapeutic strategies based on pyroptosis in NAFLD.

Inhibitors	Mechanism	Vitro experiment	Vivo experiment	References
MCC950	Inhibiting expression of caspase 1 and IL-1β, lowering numbers of macrophages and neutrophils in the liver	Mice	—	Mridha et al. (2017)
Glibenclamide	Inhibiting ATP-sensitive K+ channels	Rat	—	Dwivedi and Jena (2020)
CY-09	Binding to the ATP-binding motif of NLRP3 NACHT domain and inhibiting NLRP3 inflammasome activation	Mice	—	Jiang et al. (2017), Wang et al. (2021a)
Taurine	Inhibiting NLRP3 inflammasome activation	Mice	—	Qiu et al. (2018)
Silybin	Inhibiting NLRP3 inflammasome activation by NAD + /SIRT2 pathway	Mice	—	Zhang et al. (2018a)
Liraglutide	Inhibiting NLRP3 inflammasome activation	—	HepG2 cell	Yu et al. (2019)
Resveratrol	Inhibiting NLRP3 inflammasome activation	Mice	—	Yang and Lim (2014)
Verapamil	Inhibiting TXNIP/NLRP3 pathways	Mice	—	Zhou et al. (2018)
Allopurinol	Inhibiting NLRP3 inflammasome activation	Mice	-—	Wan et al. (2016)
Auranofin	Inhibiting the expressions of IL-1β, IL-18, caspase-1, and NLRP3	Mice	—	Hwangbo et al. (2020)
Obeticholic acid	Inhibiting NLRP3 inflammasome activation	Mice	BMDM, Kupffer cell, BMDC and LX2 cell	Huang et al. (2021)
Exenatide	Inhibiting the NLRP3 inflammasome	Mice	—	Shao et al. (2018)
Praliciguat	Inhibiting the priming (expression of Il1b and NLRP3) and blocking the release of mature IL-1β	—	Kupffer cell	Flores-Costa et al. (2020)
Berberine	Inhibiting NLRP3 and GSDMD-N expression as well as caspase-1 activity	Mice	AML12 cell	Mai et al. (2020)
Demethylenetetrahydroberberine (DMTHB)	Inhibiting NLRP3 inflammasome signaling	Mice	L02 cell	Zhang et al. (2021b)
Naringenin	Inhibiting activation of the NLRP3/NF-κB pathway and expression of NLRP3	Mice	HepG2 cell	Wang et al. (2020)
Lycium barbarum polysaccharides (LBP)	Inhibiting NLRP3/6 inflammasome pathway and NF-κB activation	Mice	—	Xiao et al. (2018)
Salidroside	Inhibiting NLRP3 inflammasome activation	Mice	—	Zheng et al. (2018)
4-acetylantroquinonol B (4-AAQB)	Inhibiting NLRP3 inflammasome activation	Mice	RAW264.7, J774A cell	Yen et al. (2021)
Dieckol (DK)	Inhibiting the NLRP3 inflammasome	Mice	—	Oh et al. (2021)
Glycyrrhizin (GL)	Inhibiting NLRP3 inflammasome activation	Mice	—	Yan et al. (2018)
Salvianolic acid A (SalA)	Inhibiting NLRP3 inflammasome activation	Rat	—	Ding et al. (2016)
Ginseng saponin	Inhibiting NLRP3 inflammasome activation	Mice	—	Wang et al. (2021b)
Magnolol	Inhibiting NLRP3 inflammasome activation	Rat	HepG2 cell	Kuo et al. (2020)
Apigenin (API)	Inhibiting NLRP3 inflammasome activation and release of inflammatory cytokines IL-1β and IL-18	Mice	Hepatic cell	Lv et al. (2019)
2,3,5,4'-tetrahydroxy-stilbene-2-O-β-D-glucoside (TSG)	Inhibiting the expression of ASC and caspase-1	Mice	—	Han et al. (2018)
Fufang zhenzhu tiaozhi	Inhibiting NLRP3 inflammasome formation and activation	Mice	Hepatic stellate cells (HSCs)	Chen et al. (2018)
Chaihu Shugan San	Inhibiting NLRP3 inflammasome pathway	Rat	—	Liang et al. (2018)
Benzyl isothiocyanate	Inhibiting cathepsin B release from lysosomes and binding to NLRP3	Mice	—	Chen et al. (2020)
Gegen Qinlian Decoction	Blocking the expression of NLRP3, ASC and caspase-1	Rat	—	Ying et al. (2021)
Sulforaphane (SFN)	Inhibiting NLRP3 inflammasome activation	Mice	—	Yang et al. (2016)
Antrodia cinnamomea	Inhibiting NLRP3 inflammasome activation	Mice	RAW264.7, HepG2 cell	Yen et al. (2020)
Cannabidiol	Inhibiting the NF-κB p65 nuclear translocation and the activation of NLRP3 inflammasome	Mice	RAW264.7 cell	Huang et al. (2019)
Sweroside	Inhibiting activation of NLRP3 inflammasome	Mice	macrophages and liver cell	Yang et al. (2020)
Jiangzhi Ligan Decoction	Inhibiting the expression of IL-1β, IL-18, TNF-α, and IL-6	Rats	—	Yang et al. (2020)
Trilobatin	Inhibiting expression of NLRP3, p65, NF-κB, cleaved-Caspase-1 and N-GSDMD, as well as the release of IL-18 and IL-1β	Mice	—	Zhang et al. (2022)
Vitamin D	Inhibiting activation of NLRP3 inflammasome	Rat	—	Zhang et al. (2021c)
Baicalin	Inhibiting expression of NLRP3, GSDMD and IL-1β expression	—	HepG2 cell	Shi et al. (2020)
Poria cocos polysaccharides (PCP)	Regulating PARP-1	Mice	—	Ye et al. (2022)
Benzyl isothiocyanate	Inhibiting the NLRP3 inflammasome activation by enhancing the PKA-dependent ubiquitination of NLRP3	Mice	Kupffer cells and hepatocytes	Lo et al. (2023)

## 5 Conclusion

Over the past few decades, researchers have conducted numerous studies to elucidate the pathogenesis of NAFLD in order to enhance its prognosis. Among these investigations, pyroptosis has been identified as playing a significant role in the initiation and progression of NAFLD. This review focused on delineating the functions of key molecules involved in cellular pyroptosis, such as the NLRP3 inflammasome, GSDMD, the caspase family, and their roles in mediating pyroptosis during the development of NAFLD. Additionally, the review discussed the innovative therapeutic strategies currently under exploration and outlines future prospects for development in this area. Nevertheless, the specific mechanisms underlying cellular pyroptosis in NAFLD remain ambiguous. The degree to which NLRP3 inflammasome activation contributes to pyroptosis is not fully elucidated. Additionally, various molecular agents targeting the NLRP3 inflammasome and GSDMD have been identified, showing promising therapeutic potential. However, these pharmaceuticals are yet to receive approval from regulatory bodies like the Food and Drug Administration (FDA). Hence, further research is imperative to unravel the precise involvement of cellular pyroptosis in NAFLD, establishing a more comprehensive theoretical foundation for future targeted therapeutic interventions.
